# High Glucose Shifts the Oxylipin Profiles in the Astrocytes towards Pro-Inflammatory States

**DOI:** 10.3390/metabo11050311

**Published:** 2021-05-13

**Authors:** Dmitry V. Chistyakov, Sergei V. Goriainov, Alina A. Astakhova, Marina G. Sergeeva

**Affiliations:** 1 Belozersky Institute of Physico-Chemical Biology, Lomonosov Moscow State University, 119992 Moscow, Russia; alina_astakhova@belozersky.msu.ru (A.A.A.); mg.sergeeva@gmail.com (M.G.S.); 2 SREC PFUR Peoples’ Friendship University of Russia (RUDN University), 117198 Moscow, Russia; goryainovs@list.ru

**Keywords:** astrocytes, high glucose, inflammation, interleukin 6 (IL-6), oxylipins, eicosanoids, toll-like receptors (TLRs), tolerance

## Abstract

Hyperglycemia is associated with several complications in the brain, which are also characterized by inflammatory conditions. Astrocytes are responsible for glucose metabolism in the brain and are also important participants of inflammatory responses. Oxylipins are lipid mediators, derived from the metabolism of polyunsaturated fatty acids (PUFAs) and are generally considered to be a link between metabolic and inflammatory processes. High glucose exposure causes astrocyte dysregulation, but its effects on the metabolism of oxylipins are relatively unknown and therefore, constituted the focus of our work. We used normal glucose (NG, 5.5 mM) vs. high glucose (HG, 25 mM) feeding media in primary rat astrocytes-enriched cultures and measured the extracellular release of oxylipins (UPLC-MS/MS) in response to lipopolysaccharide (LPS). The sensitivity of HG and NG growing astrocytes in oxylipin synthesis for various serum concentrations was also tested. Our data reveal shifts towards pro-inflammatory states in HG non-stimulated cells: an increase in the amounts of free PUFAs, including arachidonic (AA), docosahexaenoic (DHA) and eicosapentaenoic (EPA) acids, and cyclooxygenase (COX) mediated metabolites. Astrocytes cultivated in HG showed a tolerance to the LPS, and an imbalance between inflammatory cytokine (IL-6) and oxylipins release. These results suggest a regulation of COX-mediated oxylipin synthesis in astrocytes as a potential new target in treating brain impairment associated with hyperglycemia.

## 1. Introduction

Oxylipins represent a superfamily of bioactive lipid mediators, derived from the metabolism of polyunsaturated fatty acids (PUFAs), through a complex network of biochemical reactions [1,2,3]. Oxylipins have fundamental roles in a diverse set of homeostatic and inflammatory processes, and therefore, their synthesis is under intensive consideration. The conversion of PUFAs into oxylipins occurs via four major pathways, involving cyclooxygenases (COX), lipoxygenases (LOX), and cytochrome P450 monooxygenases (CYP450), and non-enzymatically [1,4,5]. When cells are stimulated, it is important that different PUFAs, and even some of their derivatives, are released from membrane phospholipids and metabolized into oxylipins simultaneously [6,7]. Therefore, there is a competition between pathways for their substrates [1,4]. Essential for oxylipin synthesis, PUFAs are subdivided into omega-3 (n-3) and omega-6 (n-6) groups; docosahexaenoic acid (DHA) and eicosapentaenoic acid (EPA) belong to n-3, and linoleic acid (LA) and arachidonic acid (AA) belong to the n-6 group. An imbalance between n-3 and n-6 PUFAs is generally attributed to chronic inflammation [8,9], although the system of oxylipins synthesis is currently viewed as being more complicated [3,10]. For example, the oxylipins of the n-6 group include both pro-inflammatory compounds and resolution substances, which are responsible for restoring the system after the pro-inflammatory stimulus has been applied [2,3,10]. The complexity of the oxylipin metabolism system has led to the development of mass spectrometric detection methods, making it possible to simultaneously measure several tens of oxylipins, derivatives of various PUFAs [11,12,13]. The measurement of oxylipin profiles reveals new steps in understanding the role of oxylipins in various pathologies.

Astrocytes are glial cells that fulfill a large number of various homeostatic maintenance functions and also play an active role in neuroinflammation, and therefore have a critical contribution to neurological disorders, including neurodegenerative and demyelinating diseases, epilepsy, trauma, ischemia, infection, and cancer [14,15,16]. Understanding astrocyte biology at the cellular and molecular levels is the cornerstone of modern research [17]. As a regulator of brain inflammation, astrocytes can release various immune and inflammatory mediators, such as pro- and anti-inflammatory cytokines/chemokines and oxylipins [3,16,18]. The data reveal that oxylipin profiles associate with different states of polarization to generate a pro-inflammatory or anti-inflammatory phenotype [19]. This association manifests itself both in native cells and in their responses to a pro-inflammatory stimulus. The data open an approach for the regulation of astrocyte function via modulation of oxylipin synthesis.

It is known that hyperglycemia, as a state of increased glucose concentrations in body fluids, causes neurotoxicity and other cerebral complications [20,21]. Patients with diabetes exhibit symptoms of neuroinflammation, cognitive impairment and neurodegenerative disorders [22,23,24].

It was shown that changes in glucose levels play an important role in the development of various pathologies, including epilepsy, which could be addressed by using ketogenic diets to limit carbohydrate intake [25,26]. Indeed, abnormal glucose levels, either too high or too low, can cause seizures [27]. Epilepsy was associated with glycogen in the brain and, therefore, with glucose levels [28]. Increased levels of ketone bodies in ketosis can also be achieved by altering blood glucose levels [26]. The availability of glycogen contributes to maintaining the proper balance between excitatory and inhibitory neurotransmission [28]. Thus, consumption, storage of glucose, acute reaction to changes, or adaptation of glucose in long-term applications play an important role in brain function and pathology. It became obvious that these processes change not only energy consumption but also the relationships between different systems, primarily the metabolic and innate immune systems.

Besides their special roles in neuroinflammation [14,16], astrocytes are the primary contributors to glucose metabolism in the brain [29]. In the brain glycogen is present mainly in astrocytes [28,29,30]. It is not surprising that these cells contain three types of peroxisome proliferator-activated receptors (PPARs) with interconnecting regulatory processes between them [31]. PPARs are transcription factors that play an important role in various metabolic processes, including glucose production [32]. Astrocyte functions are regulated by PPAR receptor agonists and antagonists [33]. It was shown that cell culturing rat brain astrocytes for 2 days in a medium with a high glucose concentration (25 mM) modulated the expression of PPARs (a decrease in PPARα and PPARβ mRNA levels without changing the PPARγ mRNA level) [34]. Although it is known that oxylipins activate PPAR [32], the dependence of their synthesis on glucose concentration has not been previously evaluated.

At present, various experimental cellular models of hyperglycemia in astrocytes have been suggested [35,36,37]. An impairment of pro-inflammatory cytokines’ release and oxidative/nitrosative stress was reported [34,36,37]. Data support the assumption that glucose is a direct modulator of changes in those functions of astrocytes that are associated with inflammatory processes. It was shown that a transcription factor, NF-kB, and mitogen-activated protein kinase (MAPK) p38 and JNK, are involved in the glucose-induced glial toxicity [34,36]. Both in vivo models of hyperglycemia and in vitro models of long-term cultivation of cells in a high glucose (HG) medium demonstrated an impairment of astrocyte functions, such as a decrement in gap junctional communication or oxidative stress induction [35,36,38]. Thus, the relationship between hyperglycemia and disturbances in the innate immune system has been shown; however, it is unknown how the synthesis of oxylipins is involved in these processes. Therefore, we compared the effect of HG on the oxylipin release in rat primary astrocytes and their ability to respond to a lipopolysaccharide (LPS) stimulation.

## 2. Results

### 2.1. Changes in the Oxylipin Profiles in Astrocytes, Cultured in Normal and High Glucose

**Figure 1:**
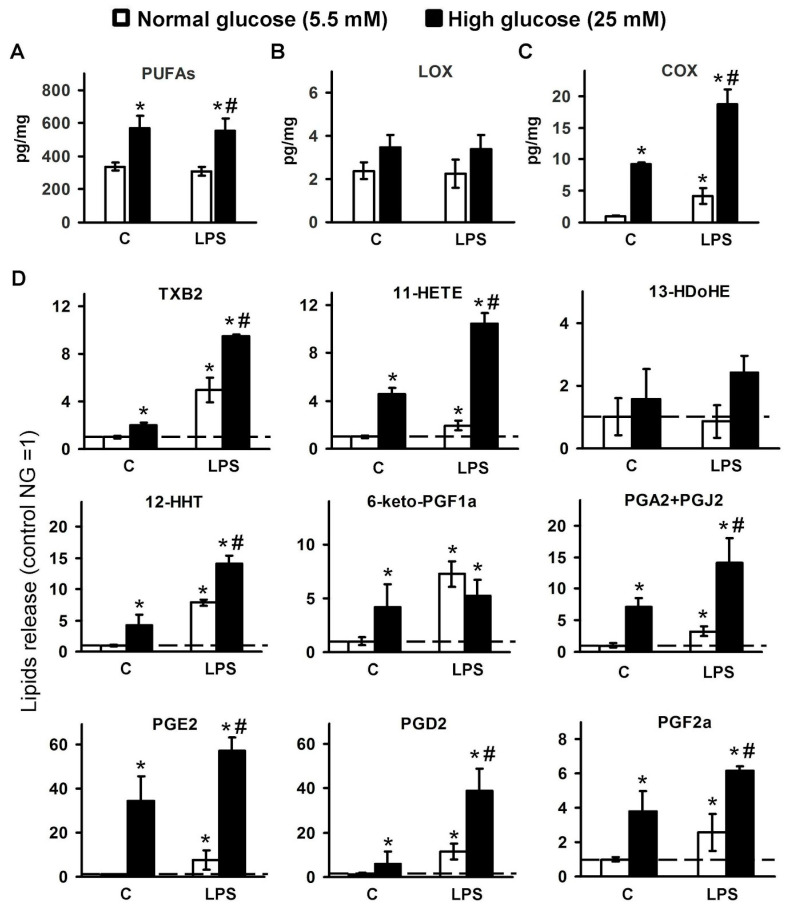
Dependence of detected oxylipins on glucose concentrations in the culture medium in LPS-stimulated cells cultivated in normal (5.5 mM) or high (25 mM) glucose medium. Primary rat astrocytes were cultivated in normal (5.5 mM) or high (25 mM) glucose medium and then stimulated with lipopolysaccharide (LPS, 100 ng/mL) for 4 h. Concentrations of oxylipins in the supernatants were measured using ultra-performance liquid chromatography-tandem mass spectrometry (UPLC-MS/MS). The values represent a mean ± SEM from three independent experiments. (**A**) the concentrations of the detected PUFAs were summed up; (**B**) the concentrations of the detected LOX-derived metabolites were summed up; (**C**) the concentrations of the detected COX-derived metabolites were summed up; (**D**) the results expressed as a fold change, relative to the naive cells, incubated in 5.5 mM glucose medium. * *p* < 0.05, compared with the unstimulated cells under normal glucose conditions, # *p* < 0.05, compared with the LPS-stimulated cells under normal glucose conditions. Abbreviations: C-control, PUFAs-polyunsaturated fatty acids, LOX-lipoxygenase, COX-cyclooxygenase, TXB2-thromboxane B2, HETE-hydroxyeicosatetraenoic acids, HDoHE-hydroxydocosahexaenoic acids, HHT-hydroxyheptadecatrenoic acid, 6-keto-PGF1a-6-keto-prostaglandin F1a, PGA2-prostaglandin A2, PGJ2-prostaglandin J2, PGE2-prostaglandin E2, PGD2-prostaglandin D2, PGF2a-Prostaglandin F2a.

To compare oxylipin profiles, primary rat astrocytes were cultured in medium with normal (5.5 mM) or high (25 mM) glucose concentrations. In the experiment, cells were stimulated with lipopolysaccharide (LPS, 100 ng/mL) for 4 h. Oxylipins in supernatants were extracted and measured using ultra-performance liquid chromatography-tandem mass spectrometry (UPLC-MS/MS). We obtained oxylipin profiles for four groups: (1) normal (NG) naive cells; (2) high (HG) glucose naive cells; (3) NG cells, stimulated by LPS; (4) HG cells, stimulated by LPS (Figure 1). For details of data, see Supplementary Table S1.

**Figure 2:**
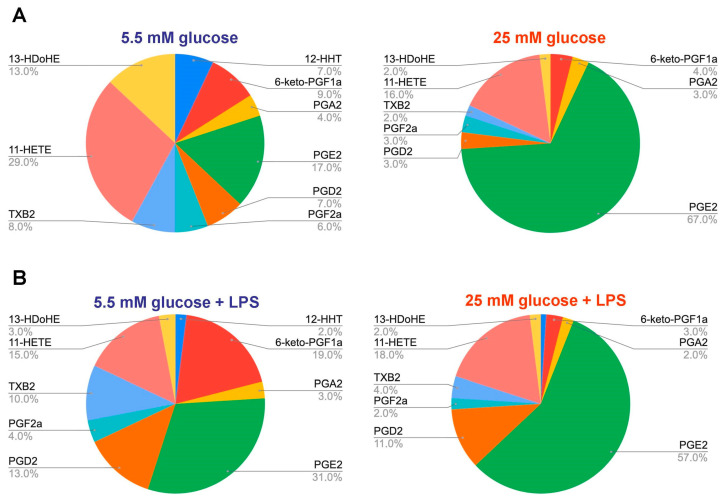
Comparison of COX-mediated metabolites in astrocytes, cultivated in normal (5.5 mM) and high (25 mM) glucose medium. Primary rat astrocytes were cultivated in normal (5.5 mM) or high (25 mM) glucose medium and then stimulated with lipopolysaccharide (LPS, 100 ng/mL) for 4 h. Concentrations of oxylipins in supernatants were measured using ultra-performance liquid chromatography-tandem mass spectrometry (UPLC-MS/MS). Pie plots showing the distribution of COX-mediated metabolites, released from (**A**) naive or (**B**) LPS-stimulated astrocytes. Abbreviations: TXB2-thromboxane B2, HETE-hydroxyeicosatetraenoic acids, HDoHE-hydroxydocosahexaenoic acids, HHT-hydroxyheptadecatrenoic acid, 6-keto-PGF1a-6-keto-prostaglandin F1a, PGA2-prostaglandin A2, PGJ2-prostaglandin J2, PGE2-prostaglandin E2, PGD2-prostaglandin D2, PGF2a-Prostaglandin F2a.

Detected oxylipins can be subdivided into three groups: (1) free PUFAs (Figure 1A); (2) derivatives of LOX-pathway (Figure 1B); and (3) derivatives of COX-pathway (Figure 1C). The PUFAs group contains three acids: EPA, DHA, and AA. Although cell cultivation in HG increases the total content of PUFAs nearly twofold, LPS stimulation does not lead to an increase in the content of PUFAs in the medium (Figure 1A). The ratio between substances is maintained (Table S1).

Based on the previous data [3,4], we classified the following metabolites as the LOX metabolic pathway: 12- and 5-hydroxyeicosatetraenoic acids (12-HETE, 5-HETE), 9-hydroxyoctadecadienoic acid (9-HODE), 8-, 14-, and 17-hydroxydocosahexaenoic acids (8-HDoHE, 14-HDoHE, and 17-HDoHE). Cell cultivation in HG or LPS stimulation did not modulate the content of these pathway substances (Figure 1B). We detected the following derivatives of AA: 12-hydroxyheptadecatrenoic acid (12-HHT), prostaglandins (PGs) 6-keto-PGF2a, PGA2, PGE2, PGD2, PGF2a, thromboxane B2 (TXB2), 11-HETE, and also 13-HDoHE, which is a derivative of DHA. All of these oxylipins are attributed to the COX pathway of oxylipin synthesis [4,39,40]. Cell cultivation in HG increases the total content of COX derivatives (Figure 1C). The sensitivity of COX-mediated synthesis for LPS stimulation decreases for the HG group (Figure 1C). Indeed, we observed a nearly twofold increase in the concentration of COX derivatives in LPS versus naive groups for HG cells and near fourfold for NG cells (Figure 1C). Thus, although many more metabolites of the COX pathway are released at high glucose, the response to the stimulation of the pro-inflammatory stimulus became less pronounced. COX-mediated derivatives contained both pro-inflammatory and anti-inflammatory or resolution relative substances [2,3]. A detailed analysis of this group of substances reveals variations in their sensitivity for HG and LPS stimulations (Figure 1D).

For the characterization of oxylipin profiles, we compared pie plots, calculated for the COX-mediated metabolites, released from naive or LPS-stimulated astrocytes (Figure 2). The plot considers compounds regarding their concentration (%), where total COX-mediated substances concentration is taken as 100%. There is a significant change in the profile during incubation in HG, which is also notable following LPS stimulation (Figure 2). In comparison to NG/HG naive cells, there is a decrease in the proportion of a substance among others for 12-HHT, 6-keto-PGF2a, PGA2, PGD2, PGF2a, TXB2, 11-HETE, and 13-HDoHE (Figure 2). Only the proportion of PGE2 significantly increased (Figure 2). Since PGE2 is usually referred to as a marker of the inflammatory process [39], it can be assumed that there is a shift in HG towards the inflammatory process even in naive cells. For LPS-stimulated oxylipin profiles, the increase in PGE2 proportion is also notable (Figure 2). Note that a proportion of 6-keto-PGF2a decreased in LPS-stimulated oxylipin profiles for HG compared to NG (Figure 2).

### 2.2. Effect of Serum on Oxylipin Profiles in Astrocytes, Cultured at Normal and High Glucose

To further characterize oxylipin synthesis in NG and HG astrocytes, we decreased the amount of serum in the experimental culture medium and tested 10% (normal concentration for cell cultivation), 5%, and 1% of the serum. This was carried out for several reasons. Firstly, oxylipins bind with extracellular proteins [11,41], and these processes may influence oxylipin profiles and cellular responses [42,43,44]. Secondly, in our experimental protocol, HG as a stressor has a long-term application, therefore, we added a set of experiments with an acute stressor. Serum as a stressor for astrocytes was reported previously [45].

We obtained that in naive cells, in both the NG and HG groups, the serum affects the medium/cell oxylipins equilibrium (the less serum, the fewer oxylipins) (Figure S1). This effect was observed for TXB2, 11-HETE, 13-HdoHE, 12-HHT, 6-keto-PGF1a, PGA2, and PGE2. In the case of PGD2, PGE2, and PGF2a, the concentration of the compound, detected in the extracellular medium, does not depend on the serum concentration (Figure S1). Therefore, for most oxylipins, the simple assumption of the dependence of oxylipins’ concentrations on their binding with the serum proteins, works well. However, the non-dependence for three PGs reveals certain additional effects of low serum concentration. To test these assumptions, we tested how serum affects the LPS-mediated cellular response in astrocytes.

**Figure 3:**
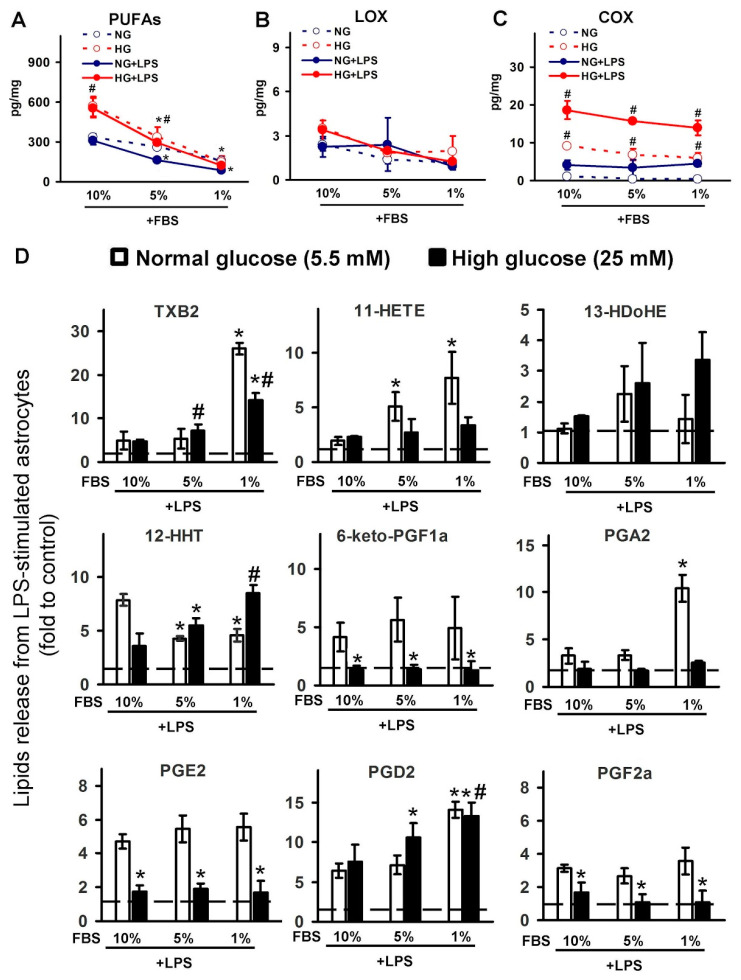
Dependence of detected oxylipins on serum concentrations in the culture medium in LPS-stimulated cells, cultivated in normal (5.5 mM) or high (25 mM) glucose medium. Primary rat astrocytes were cultivated in normal (5.5 mM) or high (25 mM) glucose medium with FBS concentration at 10%, 5%, and 1%, and then stimulated with lipopolysaccharide (LPS, 100 ng/mL) for 4 h. Concentrations of oxylipins in supernatants were measured using ultra-performance liquid chromatography-tandem mass spectrometry (UPLC-MS/MS). The values represent a mean ± SEM from three independent experiments. (**A**) the concentrations of the detected PUFAs were summed up; (**B**) the concentrations of the detected LOX-derived metabolites were summed up; (**C**) the concentrations of the detected COX-derived metabolites were summed up; (**D**) the results expressed as fold change, relative to the control, with the corresponding percentage of serum. * *p* < 0.05, compared with the LPS-stimulated cells under normal glucose conditions, with 10% FBS; # *p* < 0.05, compared with the LPS-stimulated cells under high glucose conditions, with 10% FBS. Abbreviations: PUFAs-polyunsaturated fatty acids, LOX-lipoxygenase, COX-cyclooxygenase, TXB2-thromboxane B2, HETE-hydroxyeicosatetraenoic acids, HDoHE-hydroxydocosahexaenoic acids, HHT-hydroxyheptadecatrenoic acid, 6-keto-PGF1a-6-keto-prostaglandin F1a, PGA2-prostaglandin A2, PGE2-prostaglandin E2, PGD2-prostaglandin D2, PGF2a-Prostaglandin F2a.

We obtained unexpected results when estimating the influence of serum concentration on the LPS-stimulated release of metabolites (Figure 3). During the first stage, we compared the total concentrations of metabolites, divided into PUFA, LOX-mediated, and COX-mediated metabolites. We obtained that the summarized level of PUFAs depends on the serum (in 10% FBS more than in 1% FBS) and the glucose (more in HG than in NG, with the greatest difference observed in 10% serum). Simultaneously, the stimulation of LPS cells does not significantly affect the concentration of the summarized PUFAs (Figure 3A). Cell cultivation in HG or LPS stimulation, with different serum concentrations, did not modulate the content of LOX-derived metabolites (Figure 3B). Different serum concentrations do not affect the total amount of COX metabolites, while HG increases the total level of these metabolites in all tested serum concentrations (Figure 3C).

A detailed analysis of the COX-mediated group of substances reveals variations in their sensitivity for serum, HG, and LPS stimulations. We presented the data as a “fold to a control”, which means that the synthesis of a compound in the naive cells at an appropriate serum concentration was taken as 1. A decrease in serum concentration leads to an increase in TXB2, 11-HETE, 13-HDoHE, PGA2, and PGD2. The release of 12-HHT, 6-keto-PGF2a, PGE2, and PGF2a was not dependent on serum concentration. Therefore, upon stimulation of the oxylipins synthesis by LPS, the serum acts as an additional stimulus, and a change in serum concentration affects the amount of emitted oxylipins and their ratio.

### 2.3. Effect of Serum Level and Different Glucose Concentrations in the Culture Medium on the IL-6 Release

For further characterization of the inflammatory response in astrocytes cultivated in NG and HG, we compared the release of IL-6 in LPS-stimulated cells in the presence of 10% and 1% of serum. It is known that the synthesis and release of IL-6 were at a detectable level only in the presence of LPS, not in the naive cells [46,47], therefore it can be used as a marker of astrocyte activation upon stimulation.

**Figure 4:**
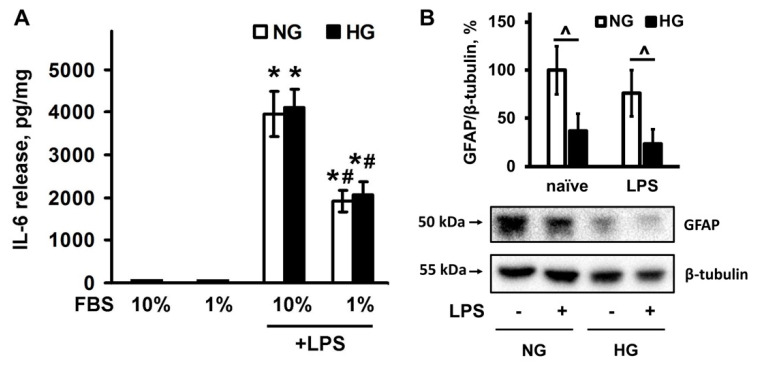
Dependence of GFAP expression and IL-6 release from serum concentration in the culture medium in LPS-stimulated cells, cultivated in normal (NG, 5 mM) or high (HG, 25 mM) glucose medium. (**A**) Primary rat astrocytes were cultivated in NG or HG medium with FBS concentration at 10% or 1%, and then stimulated with lipopolysaccharide (LPS, 100 ng/mL) for 4 h. IL-6 protein release was measured by ELISA in supernatant samples. The results are expressed as pg/mg. (**B**) primary rat astrocytes were cultivated in NG or HG medium with FBS concentration at 10%, and then stimulated with LPS, 100 ng/mL) for 4 h. Protein levels were evaluated by western blotting and normalized to the loading control β-tubulin. The values represent a mean ± SEM from three independent experiments. * *p* < 0.05, compared with the naive cells, # *p* < 0.05, compared with the LPS-stimulated cells with 10% FBS, ^ *p* < 0.05 compared with the indicated bars. Abbreviations: GFAP-glial fibrillary acidic protein, FBS-bovine fetal serum, IL-interleukin.

There is no difference in IL-6 release between cells, cultivated in NG and HG (Figure 4A). A decrease in serum concentration resulted in a nearly twofold increase in LPS-stimulated IL-6 release, although there was no difference between the NG and HG groups (Figure 4A). Thus, culturing cells in HG does not change the release of the pro-inflammatory cytokine, i.e., the work of the ensemble of the cell response to the pro-inflammatory stimulus is impaired (the ratio between the released cytokines and oxylipins).

Glial fibrillary acidic protein (GFAP) formed part of the cytoskeleton of mature astrocytes and other glial cells and was used as a marker of injured astrocytes [14]. To characterize the differences between the NG and HG groups, we measured the level of GFAP expression (Figure 4B). Expression levels of GFAP decreased in the HG group compared to the NG group (Figure 4B). Both groups did not respond to 4 h LPS stimulation by altering the level of GFAP expression (Figure 4B).

## 3. Discussion

Our data reveal that astrocyte cultivation in the high concentration of glucose (25 mM) demonstrated impairment in the innate immune system statements. Compared with astrocytes cultivated in a normal concentration of glucose (5.5 mM), cells from an HG cultivation medium exhibit the following properties: (1) an inflamed state of the naive cells measured by oxylipins levels; (2) an imbalance between the pro-inflammatory cytokine (IL-6) and oxylipins’ release; and (3) a tolerance in the oxylipins’ synthesis response to the pro-inflammatory stimulus, LPS.

It has been known for more than a decade that metabolism and immunity are closely linked, and lipids play an important role in this link [48]; however, molecular mechanisms are still largely unknown. Primary cell cultures are extensively used to facilitate the study of the molecular mechanisms involved in various physiological and pathophysiological processes. Astrocyte-enriched cultures are usually used for glial cell response studies [49]. Although Dulbecco’s Modified Eagle’s Standard Medium (DMEM) contains 25 mM or 5.5 mM glucose, glucose concentration in the culture medium used is often not indicated. Supplementation with 5.5 mM glucose in culture media approximates normal blood glucose levels in vivo, glucose levels approaching 10 mM are close to pre-diabetic levels, whereas 25 mM is considered to be a diabetic-like condition. Relatively recently, it became clear that the glucose concentration differences can affect intracellular signaling responses in various cell types (for example, [50]), including glial cells [51,52].

Various glucose applications have been tested for astrocytes: short-term (study of the glucose effect for several hours) [36,37], medium-term (48 h) [34,37], and long-term (two or more weeks of cultivation from cellular preparation) [35,51,52]. The data in the short-term glucose stimulation demonstrate the involvement of innate immune signaling pathways in the response, such as the activation of NF-kB and p38 MAPK, and pro-inflammatory cytokines’ release [36,37,38]. Such glucose effects may induce cellular responses of adaptation. The data relating to experiments with a middle-term application of high glucose concentration are in accordance with this assumption. Indeed, HG enhanced the LPS-stimulated pro-inflammatory cytokine release, increased p38, decreased JNK MAPK activities, and increased PGE2 release [34]. The long-term cultivation of cells in an HG medium resulted in the impairment of gap junctional communication [35], metabolic efficiency and capacity [53], activated the AMP-activated protein kinase (AMPK) signaling pathway, increased glycogen storage and ATP, and decreased proliferation [51]. One study claims that long-term HG cultivation reduces the ability of astrocytes to respond to LPS, but this phenomenon has not been studied in detail [52]. Interestingly, a decrease in glucose concentration below the normal level (2 mM) also induces the pro-inflammatory responses on astrocytes, manifested by increased pro-inflammatory cytokines’ release [54]. It is known that changes in the glucose transporter’s expression and functions correlate with diseases in the brain [55], which are also attributed to diseases with neuroinflammatory complications. Our data and those of others indicate that the relationship between glucose metabolism and inflammatory responses is related to maintaining the homeostasis system in the brain, and its changes are manifested in various pathologies.

It is important to note that it is currently difficult to assess the intracellular mechanisms of glucose modulation of the cellular response to inflammatory stimuli, firstly because of the differences in the experimental protocols, and secondly because of the multiple reactivities of cells to extracellular glucose concentrations. Differing glucose addition times and media change protocols can affect the readings. For example, we found that GFAP levels depend only on glucose concentration and not on LPS stimulation. The absence of changes after stimulation can be explained by the short time of action of LPS (4 h); the data are consistent with others [56]. A change in GFAP level was found 24 h or more after stimulation with LPS [37,57]. From another point of view, it should be borne in mind that the protocol in Wang’s work with colleges [37] included the stage of processing cells without glucose in the medium, which may be the reason for the difference in the obtained data.

The synthesis of oxylipins at the cellular level and the mechanism of their action via special receptors is a complex system. Oxylipins are generated by complex enzymatic reactions that begin with PUFA release from membrane phospholipids by phospholipase A2 (PLA2) [7,40]. It is known that various PLA2s are responsible for the release of AA and DHA in astrocytes [58]. Some of the oxylipins can be included in the composition of phospholipids and released by them [6]. Therefore, it is difficult to directly relate solely the oxylipins released into the extracellular medium, with their enzymatic synthesis. The scarcity of data concerning cellular oxylipin synthesis does not identify the connection between PUFAs and oxylipin profiles in the naive cells, cultivated in HG and NG with intracellular processes of oxylipin synthesis. For COX-mediated branches of metabolism, we detected, besides PGs and Tx, the following derivatives of AA: 12-HHT, 11-HETE, and also 13-HDoHE, which is a derivative of DHA. Note that DHA works as an inhibitor for COX-1 [59] and a substrate for COX-2 [60]. In addition, PGF2a may be the result of several enzyme activities: PGF synthase (from PGH2), PGD211 keto-reductase (from PGD2), and PGE2 9 keto-reductase (from PGE2) [61]. 12-HHT may result from the Tx synthase pathway or non-enzymatically from PGH2. Therefore, its synthesis is solely dependent on COX activities [62]. An elucidation of the reasons for changes in oxylipin profiles at the level of intracellular oxylipin metabolism is the subject of further research. Our data reveal the tune regulation of oxylipin synthesis and its significant dependence on glucose metabolism in astrocytes.

The observed increase in the synthesis of prostaglandins at high glucose levels is in good agreement with the data relating to a decrease in the proliferation of astrocytes [51] and the data on the summation of the effects of prostaglandins in suppressing proliferation [63]. Our data support the view of astrocytes as key cells at the crossroad of metabolic and inflammatory pathways.

## 4. Materials and Methods

### 4.1. Reagents

Lipopolysaccharide (cat.no. L2630) was from Sigma-Aldrich, St. Louis, MO, USA, ML355 (cat.no. 18537). Streptomycin–penicillin (cat.no. A063), trypsin (cat.no. P037), EDTA, fetal bovine serum (cat.no BS-110/500) were from PanEco, Moscow, Russia. Culture medium Dulbecco’s Modified Eagle Medium (DMEM) (1 g/L glucose cat.no. 21885-025) and Dulbecco’s Modified Eagle Medium (DMEM) (4.5 g/L glucose cat.no. 41966-029) were from Gibco, Thermo Fisher Scientific, Waltham, MA, USA. ELISA kit for IL-6 (cat.no. 550319) was from BD Biosciences, San Diego, CA, USA. Antibodies against β-Tubulin (D3U1W) #86298 were from Cell Signaling Technology, Danvers, MA, USA and Glial Fibrillary Acidic Protein (GFAP) M0761 was from DAKO Agilent, Santa Clara, CA, USA. Secondary horseradish peroxidase conjugated antibodies (anti-mouse) (Cell Signaling Technology, Danvers, MA, USA) and western blotting substrate ECL (Thermo Fisher Scientific, cat.no 32209, Waltham, MA, USA) were used. The oxylipins standards were as follows: 6-keto PGF1α-d4 (cat.no. 315210), TXB2-d4 (cat.no. 319030), PGF2α-d4 (cat.no. 316010), PGE2-d4 (cat.no. 314010), PGD2-d4 (cat.no. 312010), 5(S)-HETE-d8 (cat.no. 334230), 12(S)-HETE-d8 (cat.no. 334570), 15(S)-HETE-d8 (cat.no. 334720), EPA-d5 (cat.no, 10005056), DHA-d5 (cat.no. 10005057), AA-d8 (cat. No. 390010) and were from Cayman Chemical, Ann Arbor, MI, USA. Oasis^®^ PRIME HLB cartridges (60 mg, 3cc, cat.no. 186008056) were obtained from Waters, Eschborn, Germany.

### 4.2. Primary Astrocyte Cell Culture

Astrocyte-enriched cultures were obtained from newborn rats of both sexes as previously reported [34,58]. More than 95% of the cells were positive for the astrocyte marker glial fibrillary acidic protein in these cultures, and only <2% were positive for a microglia-specific marker. In brief, brains from newborn rats were triturated by the use of nylon meshes of 250 and 136 mL pore width, in consecutive order. Dissociated cells were plated into culture flasks. The cells were subsequently cultured in DMEM (1 g/L D-glucose or 4.5 g/L D-glucose, 10% bovine fetal serum (FBS), 50 units/mL streptomycin, 50 μg/mL penicillin) at 37 °C, with 10% CO_2_. After 5 days of cultivation in DMEM, the culture medium was replaced with a fresh medium, and flasks were placed on a shaker at 200 RPM for 4 h to dissociate microglial cells. The culture medium was changed to a fresh one. After 2 days, the monolayer of astrocytes was trypsinized and plated into six-well plates and maintained for 2 days in DMEM. After this, the medium was changed, and the cells were used for experiments. At each change of medium, the cells were cultured only with the glucose concentration upon their preparation. This was performed to eliminate the possible reaction of cells to changes in glucose concentration. After the cell experiments, the supernatant was collected and stored with 0.005% BHT at −80 °C for further analysis.

### 4.3. UPLC-MS/MS Conditions and Sample Preparation

After the cell experiments, the supernatant was collected and stored at −80 °C for further analysis. The cell-free culture media were taken for the solid-phase lipid extraction (Oasis^®^PRIME HLB cartridge (60 mg, 3 cc)). The cartridge was washed with 2 mL of 15% methanol containing 0.1% formic acid, and the lipids were sequentially eluted with 500 μL of anhydrous methanol and 500 μL of acetonitrile. The resulting samples were mixed, concentrated by the evaporation of the solvent under a gentle stream of nitrogen, and stored at −80 °C. For the identification of lipid mediators, the respective lipid extracts were analyzed using an 8040 series UPLC-MS/MS mass spectrometer (Shimadzu, Kyoto, Japan) in multiple-reaction monitoring mode at a unit mass resolution for both the precursor and product ions as described previously [63]. Briefly, the lipid compounds were separated by reverse-phase UPLC (injection volume 20 μL) using Phenomenex C8 column (2.1 mm × 150 mm × 2.6 μm) with the flow rate of 0.4 mL/min. The elution was performed using an acetonitrile gradient in 0.1% (*v*/*v*) formic acid. The target PUFAs and oxylipins were identified and quantified by comparing their MS, MS/MS, and UPLC (retention times, peak areas) data with the data obtained for deuterated internal standard compounds using Lipid Mediator Version 2 software (Shimadzu, Kyoto, Japan).

### 4.4. Western Blot Analysis

Western blot analysis was performed as described earlier [47]. In brief, cells were lysed in a radio immunoprecipitation assay (RIPA) buffer. The protein concentration was determined by the standard Bradford assay. After blocking, the PVDF membranes were subsequently subjected to a Phosphate-Buffered Saline with Tween 20 0.05%, with a respective primary antibody at 4 °C overnight with constant rotation. Secondary species-specific antibodies were applied at the concentration of 1:10,000 for 1 h at room temperature. The signals were detected using the Pierce ECL Plus Western Blotting Substrate (Thermo Scientific, Waltham, MA, USA). Mild stripping buffer (Abcam, Cambridge, UK) was used to re-probe western blot membranes. Densitometry was carried out on three different experiments. The band intensity was measured using a ChemiDoc™ XRS+ gel documentation system (Bio-Rad, Hercules, CA, USA). Densitometry was performed with ImageJ (1.51 s) software (National Institute of Health, Bethesda, MD, USA) using β-tubulin as the loading control.

### 4.5. Determination of IL-6 Enzyme-Linked Immunoassay

ELISA for IL-6 was performed using the BD OptEIA, (cat No.:550319; BD Biosciences, San Jose, CA, USA) antibody kits following the manufacturer’s instructions. Optical density was measured using a microplate reader Synergy H4 (BioTek, Winooski, VT, USA).

### 4.6. Data Analysis and Statistics

The data are expressed as mean ± SEM. The normality of data sets was assessed using the Shapiro–Wilk test. The data were subjected to a two-way ANOVA, followed by Bonferroni’s post hoc test, in order to determine the statistical significance, *p* < 0.05 was considered statistically significant. All of the experiments were repeated at least three times.

## 5. Conclusions

Our data reveal shifts towards pro-inflammatory states in HG, non-stimulated, naive cells and also a more pro-inflammatory mixture of LPS-stimulated oxylipin synthesis in HG in comparison to NG. Astrocytes cultivated in HG showed a tolerance to LPS. The data suppose a regulation of COX-mediated oxylipin synthesis as a potential new target in the treatment of brain impairment associated with hyperglycemia.

## Data Availability

Data is contained within the article or supplementary material.

## Supplementary Materials

The following are available online at https://www.mdpi.com/article/10.3390/metabo11050311/s1, Figure S1: Dependence of detected oxylipins on serum concentration in the culture medium in naive cells., Table S1: Mean +/− standard deviation of oxylipins (ng/mg) for normal glucose (5.5 mM) or high glucose (25 mM) cultivated cells.

## References

[1] Buczynski M.W., Dumlao D.S., Dennis E.A. (2009). An integrated omics analysis of eicosanoid biology. J. Lipid Res..

[2] Serhan C.N., Chiang N., Van Dyke T.E. (2008). Resolving inflammation: Dual anti-inflammatory and pro-resolution lipid mediators. Nat. Rev. Immunol..

[3] Chistyakov D.V., Astakhova A.A., Sergeeva M.G. (2018). Resolution of inflammation and mood disorders. Exp. Mol. Pathol..

[4] Gabbs M., Leng S., Devassy J.G., Monirujjaman M., Aukema H.M. (2015). Advances in Our Understanding of Oxylipins Derived from Dietary PUFAs. Adv. Nutr..

[5] Funk C.D. (2001). Prostaglandins and leukotrienes: Advances in eicosanoid biology. Science.

[6] O’Donnell V.B., Murphy R.C. (2017). Directing eicosanoid esterification into phospholipids. J. Lipid Res..

[7] Dennis E.A., Cao J., Hsu Y.H., Magrioti V., Kokotos G. (2011). Phospholipase A2 enzymes: Physical structure, biological function, disease implication, chemical inhibition, and therapeutic intervention. Chem. Rev..

[8] Scaioli E., Liverani E., Belluzzi A. (2017). The imbalance between N-6/N-3 polyunsaturated fatty acids and inflammatory bowel disease: A comprehensive review and future therapeutic perspectives. Int. J. Mol. Sci..

[9] Simopoulos A.P. (2016). An increase in the Omega-6/Omega-3 fatty acid ratio increases the risk for obesity. Nutrients.

[10] Serhan C.N. (2014). Pro-resolving lipid mediators are leads for resolution physiology. Nature.

[11] Liakh I., Pakiet A., Sledzinski T., Mika A. (2020). Methods of the analysis of oxylipins in biological samples. Molecules.

[12] Hellhake S., Meckelmann S.W., Empl M.T., Rentmeister K., Wißdorf W., Steinberg P., Schmitz O.J., Benter T., Schebb N.H. (2020). Non-targeted and targeted analysis of oxylipins in combination with charge-switch derivatization by ion mobility high-resolution mass spectrometry. Anal. Bioanal. Chem..

[13] Chhonker Y.S., Bala V., Murry D.J. (2018). Quantification of eicosanoids and their metabolites in biological matrices: A review. Bioanalysis.

[14] Escartin C., Galea E., Lakatos A., O’Callaghan J.P., Petzold G.C., Serrano-Pozo A., Steinhäuser C., Volterra A., Carmignoto G., Agarwal A. (2021). Reactive astrocyte nomenclature, definitions, and future directions. Nat. Neurosci..

[15] Liddelow S.A., Guttenplan K.A., Clarke L.E., Bennett F.C., Bohlen C.J., Schirmer L., Bennett M.L., Münch A.E., Chung W.S., Peterson T.C. (2017). Neurotoxic reactive astrocytes are induced by activated microglia. Nature.

[16] Sofroniew M.V. (2015). Astrocyte barriers to neurotoxic inflammation. Nat. Rev. Neurosci..

[17] Cunningham C., Dunne A., Lopez-Rodriguez A.B. (2019). Astrocytes: Heterogeneous and Dynamic Phenotypes in Neurodegeneration and Innate Immunity. Neuroscientist.

[18] Liddelow S.A., Barres B.A. (2017). Reactive Astrocytes: Production, Function, and Therapeutic Potential. Immunity.

[19] Chistyakov D.V., Gavrish G.E., Goriainov S.V., Chistyakov V.V., Astakhova A.A., Azbukina N.V., Sergeeva M.G. (2020). Oxylipin profiles as functional characteristics of acute inflammatory responses in astrocytes pre-treated with IL-4, IL-10, or LPS. Int. J. Mol. Sci..

[20] Tomlinson D.R., Gardiner N.J. (2008). Glucose neurotoxicity. Nat. Rev. Neurosci..

[21] Madhusudhanan J., Suresh G., Devanathan V. (2020). Neurodegeneration in type 2 diabetes: Alzheimer’s as a case study. Brain Behav..

[22] Barrett T.G., Bundey S.E., Macleod A.F. (1995). Neurodegeneration and diabetes: UK nationwide study of Wolfram (DIDMOAD) syndrome. Lancet.

[23] Chen J., Cui X., Zacharek A., Cui Y., Roberts C., Chopp M. (2011). White matter damage and the effect of matrix metalloproteinases in type 2 diabetic mice after stroke. Stroke.

[24] Ristow M. (2004). Neurodegenerative disorders associated with diabetes mellitus. J. Mol. Med..

[25] Curia G., Lucchi C., Vinet J., Gualtieri F., Marinelli C., Torsello A., Costantino L., Biagini G. (2014). Pathophysiogenesis of Mesial Temporal Lobe Epilepsy: Is Prevention of Damage Antiepileptogenic?. Curr. Med. Chem..

[26] Giordano C., Marchiò M., Timofeeva E., Biagini G. (2014). Neuroactive peptides as putative mediators of antiepileptic ketogenic diets. Front. Neurol..

[27] Stafstrom C.E. (2003). Hyperglycemia Lowers Seizure Threshold. Epilepsy Curr..

[28] Duran J., Gruart A., López-Ramos J.C., Delgado-García J.M., Guinovart J.J. (2019). Glycogen in Astrocytes and Neurons: Physiological and Pathological Aspects. Advances in Neurobiology.

[29] Bélanger M., Allaman I., Magistretti P.J. (2011). Brain energy metabolism: Focus on Astrocyte-neuron metabolic cooperation. Cell Metab..

[30] Waitt A.E., Reed L., Ransom B.R., Brown A.M. (2017). Emerging roles for glycogen in the CNS. Front. Mol. Neurosci..

[31] Aleshin S., Strokin M., Sergeeva M., Reiser G. (2013). Peroxisome proliferator-activated receptor (PPAR)β/δ, a possible nexus of PPARα- and PPARγ-dependent molecular pathways in neurodegenerative diseases: Review and novel hypotheses. Neurochem. Int..

[32] Iglesias J., Morales L., Barreto G.E. (2017). Metabolic and Inflammatory Adaptation of Reactive Astrocytes: Role of PPARs. Mol. Neurobiol..

[33] Chistyakov D.V., Astakhova A.A., Goriainov S.V., Sergeeva M.G. (2020). Comparison of PPAR ligands as modulators of resolution of inflammation, via their influence on cytokines and oxylipins release in astrocytes. Int. J. Mol. Sci..

[34] Chistyakov D.V., Azbukina N.V., Astakhova A.A., Polozhintsev A.I., Sergeeva M.G., Reiser G. (2019). Toll-like receptors control p38 and JNK MAPK signaling pathways in rat astrocytes differently, when cultured in normal or high glucose concentrations. Neurochem. Int..

[35] Gandhi G.K., Ball K.K., Cruz N.F., Dienel G.A. (2010). Hyperglycaemia and Diabetes Impair Gap Junctional Communication among Astrocytes. ASN Neuro.

[36] Quincozes-Santos A., Bobermin L.D., de Assis A.M., Gonçalves C.A., Souza D.O. (2017). Fluctuations in glucose levels induce glial toxicity with glutamatergic, oxidative and inflammatory implications. Biochim. Biophys. Acta Mol. Basis Dis..

[37] Wang J., Li G., Wang Z., Zhang X., Yao L., Wang F., Liu S., Yin J., Ling E.A., Wang L. (2012). High glucose-induced expression of inflammatory cytokines and reactive oxygen species in cultured astrocytes. Neuroscience.

[38] Hsieh H.L., Chi P.L., Lin C.C., Yang C.C., Yang C.M. (2014). Up-regulation of ROS-Dependent Matrix Metalloproteinase-9 from High-Glucose-Challenged Astrocytes Contributes to the Neuronal Apoptosis. Mol. Neurobiol..

[39] Dennis E.A., Norris P.C. (2015). Eicosanoid storm in infection and inflammation. Nat. Rev. Immunol..

[40] Christi W.W., Harwoo J.L. (2020). Oxidation of polyunsaturated fatty acids to produce lipid mediators. Essays Biochem..

[41] Maclouf J., Kindahl H., Granström E., Samuelsson B. (1980). Interactions of Prostaglandin H2 and Thromboxane A2 with Human Serum Albumin. Eur. J. Biochem..

[42] Niederstaetter L., Neuditschko B., Brunmair J., Janker L., Bileck A., Favero G.D., Gerner C. (2021). Eicosanoid content in fetal calf serum accounts for reproducibility challenges in cell culture. Biomolecules.

[43] Strokin M.L., Sergeeva M.G., Mevkh A.T. (2000). The influence of serum fatty acid binding proteins on arachidonic acid uptake by macrophages. Appl. Biochem. Biotechnol. Part A Enzym. Eng. Biotechnol..

[44] Stremmel W., Pohl J., Ring A., Herrmann T. (2001). A new concept of cellular uptake and intracellular trafficking of long-chain fatty acids. Lipids.

[45] Du F., Qian Z.M., Zhu L., Wu X.M., Qian C., Chan R., Ke Y. (2010). Purity, cell viability, expression of GFAP and bystin in astrocytes cultured by different procedures. J. Cell. Biochem..

[46] Gong P., Xu X., Shi J., Ni L., Huang Q., Xia L., Nie D., Lu X., Chen J., Shi W. (2013). Phosphorylation of mitogen- and stress-activated protein kinase-1 in astrocytic inflammation: A possible role in inhibiting production of inflammatory cytokines. PLoS ONE.

[47] Chistyakov D.V.D.V., Nikolskaya A.I., Goriainov S.V.S.V., Astakhova A.A.A.A., Sergeeva M.G.M.G. (2020). Inhibitor of hyaluronic acid synthesis 4-methylumbelliferone as an anti-inflammatory modulator of lps-mediated astrocyte responses. Int. J. Mol. Sci..

[48] Wellen K.E., Hotamisligil G.S. (2005). Inflammation, stress, and diabetes. J. Clin. Investig..

[49] Saura J. (2007). Microglial cells in astroglial cultures: A cautionary note. J. Neuroinflamm..

[50] Fonseca J., Moradi F., Valente A.J.F., Stuart J.A. (2018). Oxygen and glucose levels in cell culture media determine resveratrol’s effects on growth, hydrogen peroxide production, and mitochondrial dynamics. Antioxidants.

[51] Li W., Choudhury G.R., Winters A., Prah J., Lin W., Liu R., Yang S.H. (2018). Hyperglycemia alters astrocyte metabolism and inhibits astrocyte proliferation. Aging Dis..

[52] Hansson E., Björklund U., Skiöldebrand E., Rönnbäck L. (2018). Anti-inflammatory effects induced by pharmaceutical substances on inflammatory active brain astrocytes—Promising treatment of neuroinflammation. J. Neuroinflamm..

[53] Staricha K., Meyers N., Garvin J., Liu Q., Rarick K., Harder D., Cohen S. (2020). Effect of high glucose condition on glucose metabolism in primary astrocytes. Brain Res..

[54] Kogel V., Trinh S., Gasterich N., Beyer C., Seitz J. (2021). Long-Term Glucose Starvation Induces Inflammatory Responses and Phenotype Switch in Primary Cortical Rat Astrocytes. J. Mol. Neurosci..

[55] Koepsell H. (2020). Glucose transporters in brain in health and disease. Pflug. Arch. Eur. J. Physiol..

[56] Ladu M.J., Shah J.A., Reardon C.A., Getz G.S., Bu G., Hu J., Guo L., Van Eldik L.J. (2000). Apolipoprotein E receptors mediate the effects of β-amyloid on astrocyte cultures. J. Biol. Chem..

[57] Jang E., Kim J.-H., Lee S., Kim J.-H., Seo J.-W., Jin M., Lee M.-G., Jang I.-S., Lee W.-H., Suk K. (2013). Phenotypic Polarization of Activated Astrocytes: The Critical Role of Lipocalin-2 in the Classical Inflammatory Activation of Astrocytes. J. Immunol..

[58] Strokin M., Sergeeva M., Reiser G. (2003). Docosahexaenoic acid and arachidonic acid release in rat brain astrocytes is mediated by two separate isoforms of phospholipase A 2 and is differently regulated by cyclic AMP and Ca^2+^. Br. J. Pharmacol..

[59] Corey E.J., Shih C., Cashman J.R. (1983). Docosahexaenoic acid is a strong inhibitor of prostaglandin but not leukotriene biosynthesis. Proc. Natl. Acad. Sci. USA.

[60] Vecchio A.J., Simmons D.M., Malkowski M.G. (2010). Structural basis of fatty acid substrate binding to cyclooxygenase-2. J. Biol. Chem..

[61] Watanabe K. (2002). Prostaglandin F synthase. Prostaglandins Other Lipid Mediat..

[62] Matsunobu T., Okuno T., Yokoyama C., Yokomizo T. (2013). Thromboxane A synthase-independent production of 12-hydroxyheptadecatrienoic acid, a BLT2 ligand. J. Lipid Res..

[63] Chistyakov D.V., Grabeklis S., Goriainov S.V., Chistyakov V.V., Sergeeva M.G., Reiser G. (2018). Astrocytes synthesize primary and cyclopentenone prostaglandins that are negative regulators of their proliferation. Biochem. Biophys. Res. Commun..

