# Supplementary material for: High Glucose Shifts the Oxylipin Profiles in the Astrocytes towards Pro-Inflammatory States
Source: Metabolites. 2021 May 13;11(5):311. doi: 10.3390/metabo11050311 (PMC8152232; doi:10.3390/metabo11050311)
Supplement: Supplementary file 1 [file metabolites-11-00311-s001.zip › metabolites-1197926-SI.pdf]

Supplementary:

High glucose shifts the oxylipin profiles in the astrocytes towards pro-inflammatory states

Dmitry V. Chistyakov*[,1], Sergei V. Goriainov[2], Alina A. Astakhova[1] and Marina G. Sergeeva[1]

[1] Belozersky Institute of Physico-Chemical Biology. Lomonosov Moscow State University. Moscow 119992. Russia; alina_astakhova@yahoo.com. mg.sergeeva@gmail.com
[2] SREC PFUR Peoples' Friendship University of Russia (RUDN University). Moscow 117198. Russia; goryainovs@list.ru
* Correspondence: Chistyakof@gmail.com; Tel.: +74-95-939-4332

Table S1. Mean +/- standard deviation of oxylipins (ng/mg) for normal glucose (5.5 mM) or high glucose (25 mM) cultivated cells.

	NG cells, ng/mg	HG cells, ng/mg	NG + LPS, ng/mg	HG + LPS, ng/mg	Pathway (PUFA)
EPA	24.8±2.3	56.4±3.5	23.6±1.1	57±7.15	
DHA	306±22	490±68.6	278±25.6	477±62.8	
AA	7.34±0.14	22±2.15	7.5±0.99	22.6±3.26	
12-HHT	0.08±0.01	0.03±0.01	0.07±0.01	0.12±0.01	COX (AA)
6-keto-PGF1a	0.1±0.04	0.39±0.07	0.78±0.4	0.52±0.04	COX (AA)
PGA2	0.04±0.01	0.25±0.03	0.12±0.07	0.46±0.12	COX (AA)
PGE2	0.19±0.05	6.16±0.32	1.3±0.4	10.6±1.9	COX (AA)
PGD2	0.08±0.03	0.28±0.06	0.56±0.03	2.01±0.15	COX (AA)
PGF2a	0.07±0.01	0.25±0.05	0.16±0.05	0.4±0.06	COX (AA)
TXB2	0.09±0.01	0.18±0.01	0.43±0.15	0.83±0.06	COX (AA)
11-HETE	0.33±0.01	1.48±0.12	0.63±0.15	3.4±0.17	COX (AA)
13-HDoHE	0.15±0.09	0.19±0.01	0.11±0.01	0.3±0.02	COX (DHA)
12-HETE	0.35±0.14	0.61±0.08	0.35±0.08	0.68±0.11	LOX (AA)
5-HETE	0.27±0.06	0.43±0.06	0.23±0.05	0.36±0.01	LOX (AA)
13-HODE	0.5±0.24	0.54±0.08	0.47±0.04	0.48±0.19	LOX (LA)
13-KODE	0.07±0.05	0.02±0.02	0.07±0.06	0.06±0.02	LOX (LA)
9-HODE	0.28±0.19	0.3±0.08	0.3±0.11	0.29±0.12	LOX (LA)
9-KODE	0.08±0.06	0.12±0.06	0.06±0.03	0.07±0.04	LOX (LA)
16-HDoHE	0.19±0.07	0.19±0.06	0.1±0.05	0.15±0.04	LOX (DHA)
4-HDoHE	0.33±0.02	0.54±0.05	0.29±0.19	0.56±0.16	LOX (DHA)

8-HDoHE	0.13±0.01	0.33±0.15	0.22±0.17	0.35±0.14	LOX (DHA)
14-HDoHE	0.08±0.04	0.2±0.03	0.09±0.05	0.18±0.06	LOX (DHA)
17-HDoHE	0	0.05±0.03	0.08±0.03	0.04±0.02	LOX (DHA)
10-HDoHE	0.12±0.06	0.15±0.01	0.07±0.05	0.17±0.01	LOX (DHA)

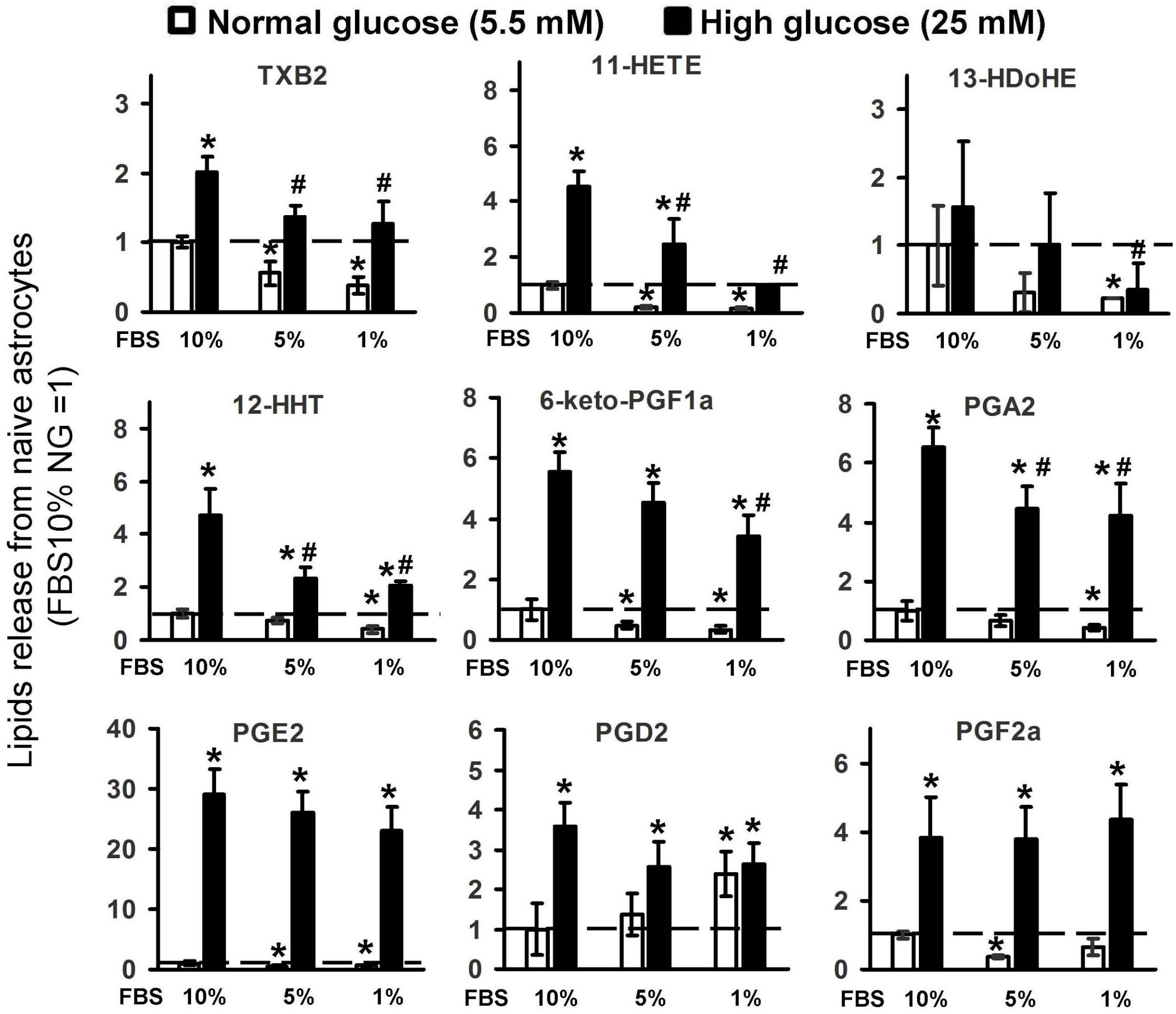

Figure S1. Dependence of detected oxylipins on serum concentration in the culture medium in naive cells. Comparison of normal (5.5 mM) and high (25 mM) glucose concentration in the cultivated medium. Primary rat astrocytes were cultivated in normal (5.5 mM) or high (25 mM) glucose medium with FBS concentration at 10%, 5%, and 1%. Concentrations of oxylipins in supernatants were measured using ultra-performance liquid chromatography-tandem mass spectrometry (UPLC-MS/MS). The values represent a mean ± SEM from three independent experiments. *p<0.05, compared with the unstimulated cells in normal glucose conditions with 10% FBS; #p<0.05, compared with the unstimulated cells in high glucose conditions with 10% FBS.
